# Effectiveness of a Non-pharmacological Intervention to Control Diabetes Mellitus in a Primary Care Setting in Kerala: A Cluster-Randomized Controlled Trial

**DOI:** 10.3389/fpubh.2021.747065

**Published:** 2021-11-16

**Authors:** Arya Rahul, Sujatha Chintha, Thekkumkara Surendran Anish, Kannamkottapilly Chandrasekharan Prajitha, Pillaveetil Sathyadas Indu

**Affiliations:** Department of Community Medicine, Government Medical College, Thiruvananthapuram, India

**Keywords:** diabetes mellitus, primary care, non-pharmacological intervention, cluster randomized controlled trial, public health workers

## Abstract

**Background:** Despite being the first Indian state with a dedicated Non-Communicable Disease (NCD) program, glycemic control among a large proportion of patients is low in Kerala. This study tries to find evidence for a standardized non-pharmacological strategy delivered through Junior Public Health Nurses (JPHNs) in achieving and maintaining glycemic control among diabetic patients registered with NCD clinics of primary health care settings.

**Design:** A cluster randomized controlled trial was conducted among adult patients with Diabetes Mellitus attending NCD clinics of primary care settings of South Kerala, India. JPHNs of the intervention group received additional module-based training while standard management continued in the control group. Sequence generation was done by random permuted blocks method and a cluster of 12 patients was selected from each of the 11 settings by computer-generated random numbers. Patients were followed up for 6 months with monthly monitoring of Fasting Blood Sugar (FBS), Post-Prandial Blood Sugar (PPBS), blood pressure, Body Mass Index (BMI), and health-related behaviors. Knowledge and skills/practice of JPHNs were also evaluated. Analysis of Covariance was done to study the final outcome adjusting for the baseline values and a model for glycemic control was predicted using multilevel modeling.

**Results:** We analyzed 72 participants in the intervention group and 60 participants in the control group according to the intention-to-treat principle. The intervention was associated with a significant reduction in FBS (*p* < 0.001) and PPBS (*p* < 0.001) adjusting for the baseline values. The achievement of glycemic control was 1.5 (95% CI: 1.05–2.3) times better with intervention and they showed a better trend of maintenance of glycemic control (FBS, *p* = 0.003 and PPBS, *p* = 0.039). Adjusting for clustering and the baseline values, the intervention showed a significant effect on FBS (B = −3.1, SE = 0.57; *p* < 0.001) and PPBS (B = −0.81, SE = 0.3; *p* < 0.001) with time. Drug adherence score (*p* < 0.001), hours of physical activity (*p* < 0.001), BMI (*p* = 0.002), fruit intake (*p* = 0.004), and green leafy vegetable intake (*p* = 0.01) were the major predictors of FBS control. The practice/skills score of the JPHNs significantly improved with intervention (*p* < 0.001) adjusting for baseline values.

**Conclusion:** A well-designed health worker intervention package incorporated into the existing health system can translate into attitude change and skill development in the health workers which can reflect in the improvement of glycemic control among the patients.

**Trial registration:** [URL: http://www.ctri.nic.in], identifier [CTRI/2017/11/010622].

## Introduction

Diabetes is a huge threat to the public health systems, at the global, national, and family levels, in terms of morbidity, mortality, and the economic burden it imposes directly and indirectly. Kerala is the state with the highest burden of diabetes in India and is the first state with a State-funded dedicated Non-Communicable Disease (NCD) Control Programme, of which control of Diabetes mellitus is a major component (1, 2). Non-communicable Disease (NCD) clinics are conducted every week at the health sub-centers (most peripheral and the first contact point between the primary health care system and the community) as a part of the NCD control program of the Government aiming at early detection, delivery of adequate health education to the patients and improving access for NCD care and drugs. However, the glycemic control among a large proportion of diabetic patients is reported to be low even among the patients who receive treatment (3–5). Understanding that primary care optimization is an effective tool toward the achievement of health system objectives (6), researches to develop contextually appropriate and resource-sensitive approaches at the primary care level become important to ensure glycemic control among patients with diabetes.

The role of non-pharmacological interventions alone and along with medications in glycemic control is well-documented elsewhere (7–10). However, there is a scarcity of evidence on the effectiveness of non-pharmacological interventions through field-level health workers, who represent an important link between patients and the health system (11). This study tries to find evidence for a standardized non-pharmacological strategy delivered through field-level health workers in improving glycemic control among known diabetic patients. The trial primarily aims to study the effectiveness of a non-pharmacological intervention delivered through Junior Public Health Nurses (JPHNs) in achieving a reduction of fasting blood glucose among diabetic patients registered with NCD clinics of primary health care settings of Southern Kerala. Secondly, changes in post-prandial blood glucose and maintenance of glycemic control were studied along with other clinical parameters like blood pressure and body mass index, and health-related behaviors. The effectiveness of standardized module-based training in improving the knowledge and skills of Junior Public Health Nurses was also studied.

## Methods

The study details and results are reported in accordance with the CONSORT statement for randomized trials of Non-Pharmacologic Treatments (NPTs) (12).

### Study Oversight

A cluster randomized control trial was conducted in the 11 health sub-centers functioning under the urban and rural health training centers of a tertiary teaching institute in south Kerala. The trial was approved by the Human ethics committee of the institute (IEC No. 12/01/2017/MCT) and was registered with the Clinical Trial Registry of India (CTRI), registration number-CTRI/2017/11/010622. The baseline data collection was done in July and August 2018 and participants were followed up for a period of 6 months.

Health sub-centers (HSC) are the most peripheral units of the health system in India and they carry out public health activities in a defined population. Each HSC in the study setting caters to a population of 7,000–11,000. The field-level health workers known as Junior Public Health Nurses (JPHNs) and Junior Health Inspectors (JHI) in Kerala are the key personnel in every HSC and are recruited through Kerala Public Service Commission. They provide comprehensive primary care services varying from promotive, preventive, curative, rehabilitative, and palliative services. The JPHNs have an educational qualification of a higher secondary degree along with an Auxillary Nurse Midwife (ANM) certificate. They discharge activities related to Maternal and Child health, communicable disease surveillance, and conduct weekly Non-Communicable Disease (NCD) clinics in the HSC. Around 50–70 patients attend the sub-center NCD clinics monthly.

### Participant Eligibility and Sample Size

Adult patients (above 18 years of age) with diabetes mellitus registered at the NCD clinics were included. Patients who were unwilling, pregnant, bedridden, suffering from malignancy, renal/hepatic disease, had cardiac events in the past year, and those with cognitive impairment were excluded. The sample size was calculated based on a study by Goldhaber et al. which reported a decrease of 19 ± 55 mg/dl in the fasting blood sugar value of the intervention group and an increase of 16 ± 78 mg/dl in the control group (13). The two-sided probability of type I error was fixed at 5% and power at 80% to calculate a minimum required sample size of 58 in each group. Since the exact intracluster correlation (ICC) between the HSCs in FBS values of patients was unknown and a high level of ICC was not expected for a non-communicable disease at the HSC level, the sample size was inflated by 10%. It was decided to enroll patients from all 11 selected HSCs as clusters of size 12.

### Randomization and Blinding

The HSCs were randomized into intervention and control groups by random permuted blocks method (block size four). The sequence generation was done by a statistician (who had no prior knowledge regarding the HSCs) and the allocation was concealed from the researchers till the day of intervention using an opaque sealed envelope. Since there were only 11 clusters for randomization, the 12th code was neglected. The eligible participants were selected from each HSC by simple random method using computer-generated random numbers. No blinding of participants was done owing to the nature of the study. Outcome assessors and data analysis was blinded.

### Intervention and Outcome Measures

#### Preparation of a Module to Train the JPHNs

A module was prepared to train JPHNs with a key focus on improving their knowledge, attitude, and skills in diabetes management at the primary health care level with due focus on a healthy diet, adequate exercise, compliance to treatment and follow-up, cessation of smoking, and alcohol intake. The module content was validated qualitatively with the help of experts by the modified Delphi technique (14). The panel included State Nodal Officer of NCD division (Government of Kerala), Deputy Director of planning (Directorate of health services), Administrative Medical Officers of the primary health centers under the study, an epidemiologist, a diabetologist, and a social scientist.

#### Content of the Training Module

The module consisted of a 1-day training to be conducted in three sessions targeting the improvement in knowledge, attitude, and skills in counseling patients with diabetes. The module was prepared in the local language, Malayalam.

1. Knowledge (Duration 1.5 h)

This session consisted of a short lecture based on andragogy principles using a PowerPoint presentation. The slides were prepared after a comprehensive literature review and expert advice regarding non-communicable disease burden, diabetes—its risk factors, complications, prevention, and control. The role of lifestyle modification in controlling diabetes was highlighted with due focus on a healthy diet, adequate physical activity, cessation of smoking and alcoholism, drug compliance, and regular follow-up.

2. Attitude (Duration 2 h)

This session involved attitude building in JPHNs through discussion of different case scenarios related to diabetes in a primary health setting. The participants were seated in a circle and a clinical scenario from the training module was read out by a participant to the group. Each participant was given a chance to discuss their views and the advice that they would offer in the scenario. The investigator acted as a facilitator for the discussion. Following this, the instructor discussed the best instructions and action plan for patients in each scenario. Flexible approaches to facilitate sustainability of behavioral changes like maintenance of a health diary, setting up of community walking groups, vegetable/fruit gardening at home, etc. were highlighted.

3. Practice/Skills (Duration 2.5 h)

This session involved hands-on training for the JPHNs simulating diabetes care in a primary care setting with diabetic patients. The participants were given two patients each and allotted a time of 30 min to gather history regarding their illness, monitor the clinical parameters, and offer counseling on diabetes care. The activities were monitored and scored by the investigators. The participants were encouraged to take feedback from the patients and identify the barriers to behavioral change. Finally, a group discussion was conducted with the JPHNs and the investigators to share their experiences and feedback from the patients. The investigators discussed the possible ways to aid effective and successful counseling through a better understanding of barriers and flexibility of approaches.

#### Training JPHNs

A 6-h training was conducted for the JPHNs in the intervention group involving discussions, role play, and hands-on sessions based on the module. Pre and post-intervention evaluation of JPHNs was done using a structured questionnaire for the assessment of knowledge and a checklist for the assessment of skills and practice.

#### Implementation at HSCs

Study participants were recruited to the respective study groups after obtaining informed consent. Their demographic and behavioral characteristics (diet, physical activity, smoking, and alcohol intake) and baseline values of blood sugar, Body Mass Index, and blood pressure were noted by the investigators. Baseline compliance to treatment was measured using the Medication Adherence Questionnaire (MAQ). A 3–5 min counseling was offered by the JPHNs of the intervention group to every patient attending the NCD clinic during their monthly visit (followed for 6 months) with a focus on diet modification, exercise, improving drug compliance, regular medical check-ups, and cessation of smoking and alcohol intake in addition to the standard treatment. The patients in the control group received standard management according to the Directorate of Health Services (Government of Kerala) guidelines (15).

#### Monitoring and Follow up

Participants were followed up for 6 months with monthly monitoring of FBS, PPBS, BMI, and Blood pressure by the JPHNs, and the values were recorded in the data collection sheets provided. Data on drug adherence, dietary patterns, physical activity, and habits of smoking and alcoholism were also collected at the end of the follow-up. All the data recorded were verified by research assistants. At the end of the trial, all patients who did not turn up for follow-up were contacted over the phone by the investigators and advised to report at the HSC on the specified date.

#### Outcomes and Definitions

All participant assessments took place at the HSC during the follow-up visits. Fasting blood sugar was the primary outcome under study. As secondary outcomes, post-prandial blood sugar, glycemic control status, and other clinical parameters like blood pressure, BMI, and health-related habits like drug adherence, dietary pattern, physical activity, and habits of smoking and alcoholism were studied. The improvement in knowledge and skills/practice of the JPHNs and patient perception of services delivered at the HSCs were also studied.

We defined glycemic control status as per the targets recommended by the American Diabetes Association (ADA) for the treatment of Diabetes (16). Controlled fasting blood sugar was defined as FBS value between 80 and 130 mg/dl and controlled postprandial blood sugar as PPBS <180 mg/dl. Achieving total glycemic control required optimum control of FBS and PPBS. Adequacy of physical activity was defined as at least 150 min of moderate-intensity physical activity throughout the week, or at least 75 min of vigorous-intensity physical activity throughout the week, or an equivalent combination of moderate and vigorous-intensity physical activity as per the World Health Organization (17). Classification of BMI was done as per WHO-recommended cut-offs for the Asian population. A BMI of 23–24.9 kg/m^2^ was considered overweight and BMI ≥ 25 kg/m^2^ was considered obesity (18). Preparation of food frequency table and quantification of food items including portion size estimation (wherever applicable) was done based on the common food materials consumed in India as reported by the Indian Council of Medical Research (National Institute of Nutrition) guidelines modified with inputs from the Kerala State Nutrition Office. Medication adherence was assessed using Morisky, Green and Levine Adherence Scale (also known as Medication Adherence Questionnaire) (19).

### Statistical Methods and Data Analysis

Statistical analysis was performed using IBM SPSS Statistics for Windows, Version 25.0. Armonk, NY: IBM Corp. Baseline characteristics of the participants were summarized using mean and standard deviation (SD) or median and interquartile range (IQR) for continuous variables and frequency and percentage for categorical variables. The primary analysis was planned to study the effect of the intervention on the FBS values at the end of 6 months adjusting for the baseline values based on the intention-to-treat principle. This was to prevent bias due to the expected missing values considering the nature of the intervention and data collection. Analysis of Covariance (ANCOVA) was used to compare the final blood sugar levels adjusting for the baseline values after assessing the assumption of linearity using residuals. The model was also adjusted for the potentially confounding sociodemographic and baseline values. Similarly, ANCOVA was used to compare the PPBS, blood pressure, and BMI values at the end of follow-up adjusting for baseline values. Glycemic control status at the end of follow-up was compared between the intervention and control group by chi-square test and the trend of glycemic control across the 6 months of follow-up was analyzed using chi-square for trend.

Multiple imputation of missing monthly glycemic values using iterative Markov Chain Monte Carlo (MCMC) method was done to create 10 imputed datasets. The imputation model was supplemented with auxiliary variables identified in preliminary analyses to be related to attrition and the pooled estimates were analyzed. Repeated measure ANOVA was done to study the change in FBS and PPBS values over the months of follow-up (Mauchy's test of sphericity indicated a violation of the assumption of sphericity and therefore a Greenhouse-Geisser correction was used).

Multilevel modeling (MLM) using the random intercepts model was done to assess the effect of the intervention over the study period on FBS and PPBS values adjusting for the clustering at the HSC level with time interaction. Adjustments for the sociodemographic and baseline variables between the two groups were also done in the model. MLM was conducted in STATA data analyses software, version 15.0. Study group (intervention or control), time point (baseline and 6 months of follow-up), and a study group-timepoint interaction were specified as fixed effects in the model. The significance of intervention was tested using the study group-time frame interaction. Intra-Class Correlation (ICC) coefficient of each model and its 95% confidence interval was calculated as the proportion of variance in the outcome that is explained by the grouping structure of the hierarchical model ($\rho =\sigma_{\text{u0}}{}^{2}/\sigma_{\text{u0}}{}^{2}+\sigma_{\text{e}}{}^{2}$ , where $\sigma_{\text{u0}}{}^{2}$ is the variance of the level-2 residuals and $\sigma_{\text{e}}{}^{2}$ is the variance of the level-1 residuals).

A model for individual factors in the intervention package as predictors of glycemic control was built adjusting for sociodemographic variables, and baseline values of drug adherence, dietary habits, physical activity, alcoholism, and smoking frequency using Generalized Estimating Equations (GEE). The post-training improvement in knowledge and skills of JPHN was assessed using ANCOVA. The threshold of statistical significance was defined at *P* < 0.05 for all analyses.

### Ethics

Written Informed consent was obtained from all study participants. The information of the participants was coded to ensure confidentiality. The trial followed good clinical practice and did not interfere with any existing treatment. Following trial completion and generation of evidence on the effectiveness of the intervention, the health workers in the control group were also given training based on the module.

## Results

### Overview of Enrolment and Data Completeness

**Figure 1:**
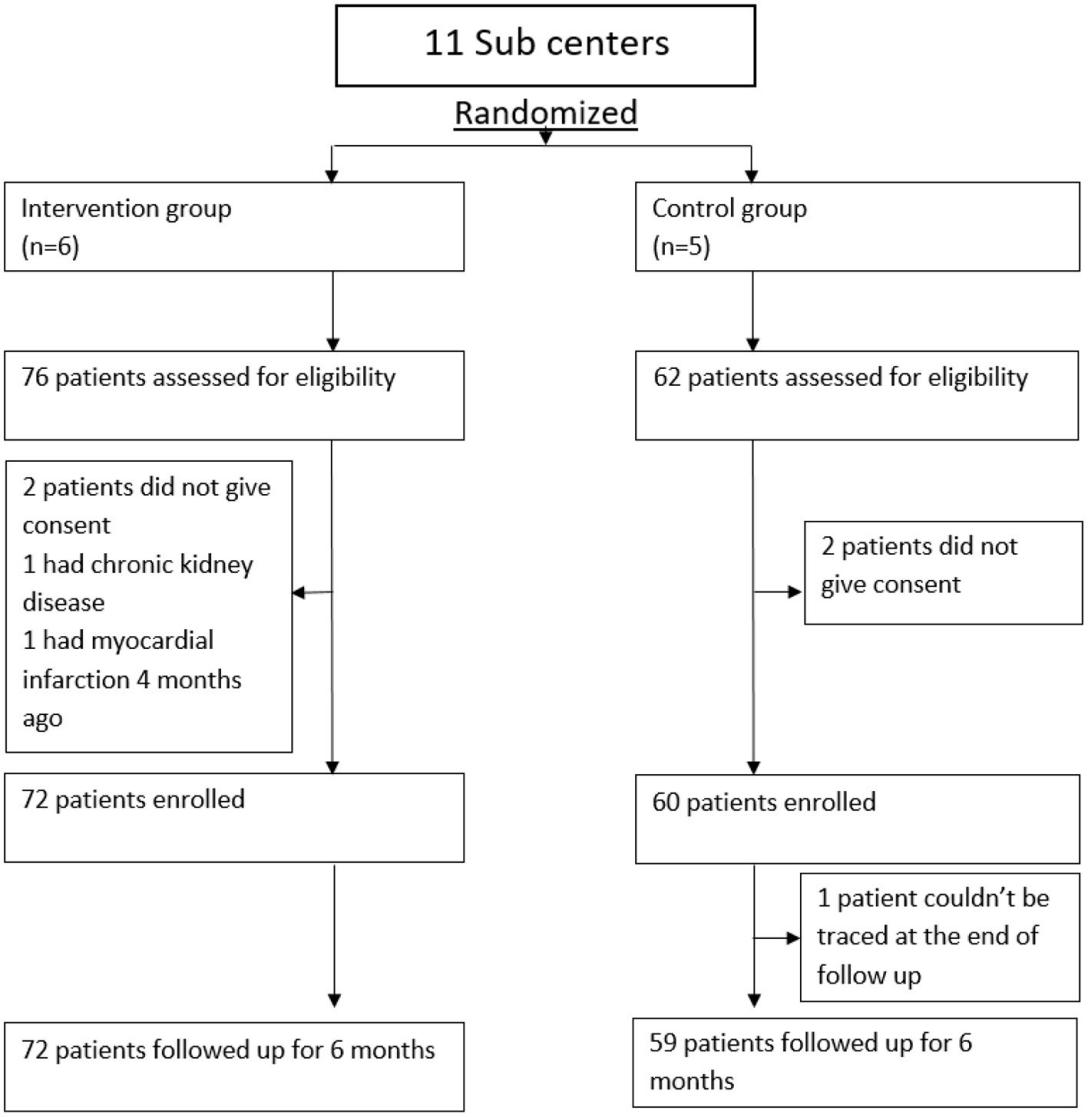
Study enrolment profile.

We recruited 72 participants to the intervention group and 60 participants to the control group and at the end of follow-up, all except one participant could be traced by the investigators (Figure 1). An intention-to-treat analysis was done including all recruited cases. Since follow-up attendance was a variable under study, the completeness of follow-up data was different in both groups and the investigators did not interfere in it.

### Baseline Characteristics of the Study Participants

Around three fourth of the participants were aged above 55 years and more than 70% were females in both groups. At the initiation of the trial, 32 (44.4%) participants in the intervention group and 32 (53.3%) participants in the control group had their FBS levels under control whereas the PPBS control was seen in 26.4 and 28.3% of participants, respectively. The baseline sociodemographic and clinical characteristics of the intervention and control group are summarized in Table 1 and the baseline dietary habits are given in Table 2. The baseline parameters in the two groups appear to be potentially imbalanced for some variables including gender, socioeconomic status, diagnosed comorbidities, blood glucose parameters, and smoking status.

**Table 1:** Baseline characteristics of the study participants.

**Variables**		**Intervention group**	**Control group**
		***n*** **= 72 (%)**	***n*** **= 60 (%)**
Age in years, mean ± SD		60.99 ± 9.6	62.77 ± 9.3
Gender	Male	21 (29.2)	11 (18.3)
	Female	51 (70.8)	49 (81.7)
Socio-economic status^*^	Lower class	4 (5.6)	1 (1.7)
	Upper lower	33 (45.8)	25 (41.7)
	Lower middle	31 (43.1)	26 (43.3)
	Upper middle	3 (4.2)	8 (13.3)
	Upper class	1 (1.4)	0 (0)
Diagnosed hypertension		57 (79.2)	51 (85)
Diagnosed hyperlipidemia		19 (26.4)	20 (33.3)
Diagnosed thyroid disease		6 (8.3)	8 (13.3)
Family history of Diabetes mellitus		46 (63.9)	41 (68.3)
Duration of Diabetes in years, median (IQR)		6 (2.5–10)	7 (4–12)
Fasting Blood Sugar (mg/dl), mean ± SD		141 ± 29	134 ± 33
Post prandial blood sugar (mg/dl), mean ± SD		218 ± 55	209 ± 57
Controlled FBS		32 (44.4)	32 (53.3)
Controlled PPBS		19 (26.4)	17 (28.3)
Controlled FBS and PPBS		17 (23.6)	15 (25)
Body Mass Index (kg/m^2^), mean ± SD		25.0 ± 4.1	25.5 ± 3.3
Systolic Blood Pressure (mm Hg), mean± SD		132 ± 15	132 ± 14
Diastolic Blood Pressure (mm Hg), mean± SD)		81 ± 7	82 ± 8
Medication adherence score, median (IQR)		1 (1–3)	1 (1–3)
Adequate physical activity		12 (16.7)	8 (13.3)
Alcohol use		6 (8.3)	5 (8.3)
Smoking		8 (11.1)	4 (6.7)
Vegetable garden at home		7 (9.7)	8 (13.3)
Fruit garden at home		8 (11)	4 (6.7)

* *Classified based on modified Kuppuswami scale*.

**Table 2:** Baseline dietary habits^#^.

**Food item**	**Mean** **±** **SD**	
	**Intervention group**	**Control group**
	***n*** **= 72**	***n*** **= 60**
Oil use (grams/day)^*^	21.1 ± 3.2	20.4 ± 3.9
Salt use (grams/day)^*^	10.9 ± 3.4	11 ± 3.1
Sugar use (grams/day)^*^	12.6 ± 4.5	11.9 ± 5
Coconut use (grams/day)^*^	86 ± 26	91 ± 30
Red meat (days/month)	0.3 ± 0.5	0.5 ± 0.7
Other meat (days/month)	1.6 ± 1	2.0 ± 1.3
Fish (days/month), median (IQR)	27 (20–30)	25 (20–30)
Egg (days/month)	4.5 ± 3.7	5.1 ± 4.1
Sweets (days/week)	0.48 ± 0.7	0.7 ± 0.8
Fried snacks (days/week)	1.2 ± 1	1.0 ± 1.0
Sugary drinks (days/week)	0.15 ± 0.3	0.3 ± 0.5
Tea/coffee (times/day)	2.2 ± 0.7	2.1 ± 0.8
Polished rice (times/day)	1.5 ± 0.5	1.6 ± 0.5
Wheat (times/day)	0.3 ± 0.5	0.3 ± 0.5
Oats/ragi (times/day)	0.2 ± 0.4	0.1 ± 0.3
Tubers (days/week)	2 ± 0.7	1.8 ± 0.9
Pickle/pappad (days/week)	1.7 ± 1.8	1.5 ± 1.6
Pulses (days/week)	2.6 ± 0.8	2.5 ± 1
Water (glasses/day)	8 ± 1.7	8.2 ± 1.5
Fruits (grams/week)	95.2 ± 70	111.7 ± 73
Green leafy vegetables (grams/week)	88.6 ± 49.3	90.3 ± 67.7
Other vegetables (grams/week)	363.9 ± 107.9	364 ± 103

# *Estimated from food frequency questionnaire*.

* *Estimated using family intake (data on standard intake per person is not available)*.

### Outcomes

At the end of 6 months, mean fasting blood sugar values dropped in both groups, the drop being higher in the intervention group. Adjusting for the baseline FBS values, FBS at the 6th month was 123.7 ± 1.71 (95% CI: 120.3–127.1) mg/dl in the intervention group and 135.4 ± 1.89 (131.6–139.1) mg/dl in the control group. The intervention was associated with a significant reduction in FBS at the end of 6-month follow-up after controlling for the effect of baseline FBS, *F*_(1,130)_ = 20.8, *p* < 0.001.

The model was further adjusted for age, gender, socioeconomic status, diagnosed comorbidities, years since diagnosis of diabetes, baseline FBS value, and smoking status. The adjusted mean 6th-month FBS value was 125.1 (±15.9) mg/dl in the intervention group and 133.6 (±23.9) mg/dl in the control group. The FBS values were significantly lower in the intervention group, *F*_(1,121)_ =14.1, *p* < 0.001, and the adjusted model showed a 10.3 (±2.6) mg/dl decline in FBS values with the intervention compared to the control group.

Adjusting for the baseline PPBS values, PPBS at the 6th month was 179.7 ± 3.59 (172.6–186.8) mg/dl in the intervention group and 210.1 ± 3.97 (202.3–217.9) mg/dl in the control group. The reduction in PPBS at the end of 6th-month follow-up was significantly better in the intervention group after controlling for the effect of baseline PPBS, *F*_(1,130)_ = 32.15, *p* < 0.001. The PPBS model adjusted for sociodemographic and other baseline variables showed a significant decline of 27.7 (±5.6) mg/dl in the PPBS values in the intervention group compared to the control group, *F*_(1,121)_ = 23.9, *p* < 0.001. The adjusted PPBS values were 182.2 (±42.2) mg/dl and 207 (±49.2) mg/dl in the intervention and control groups, respectively.

At the end of follow-up, 51 (70.8%) participants in the intervention group and 29 (49.2%) participants in the control group had their FBS under control, whereas PPBS control was present in 40 (55.6%) and 22 (37.3%) participants, respectively. The intervention was associated with a 1.73 (1.2–1.9) times incidence of FBS control and 1.6 (1.1–2.1) times incidence of PPBS control compared to the standard management. The achievement of a total glycemic control status (control of both fasting and postprandial blood sugar) was 1.5 (1.05–2.3) times higher in participants who received the intervention.

**Figure 2:**
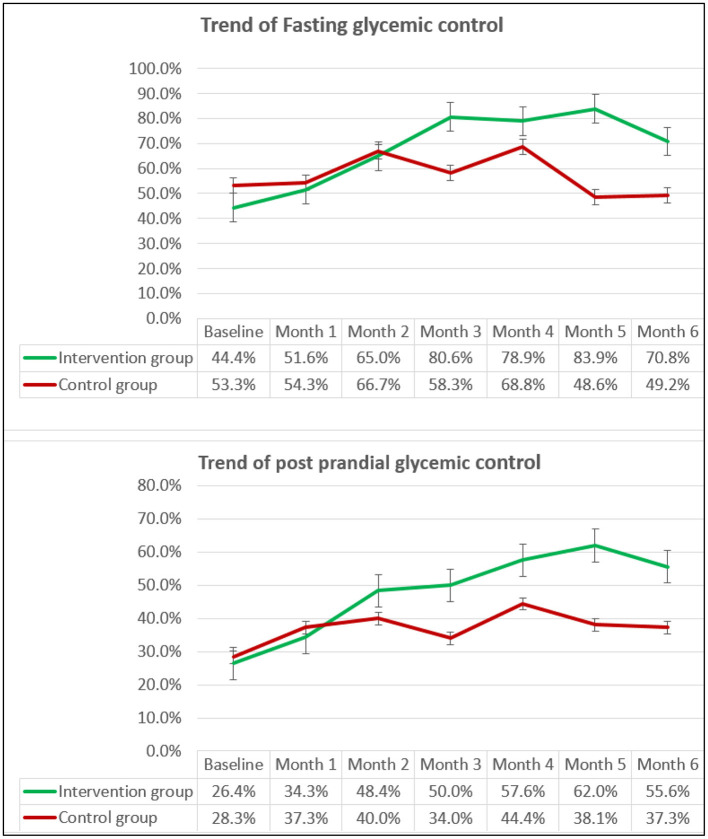
The trend of proportion^*^ of participants with Fasting and Postprandial glycemic control over the follow-up period. ^*^Percentages are calculated excluding the missing values in both groups.

The intervention group had a significantly better trend of maintenance of glycemic control (Figure 2). Chi-square value for linear trend (Extended Mantel Haenszel) was significant at *P* = 0.003 for FBS and *p* = 0.039 for PPBS.

**Figure 3:**
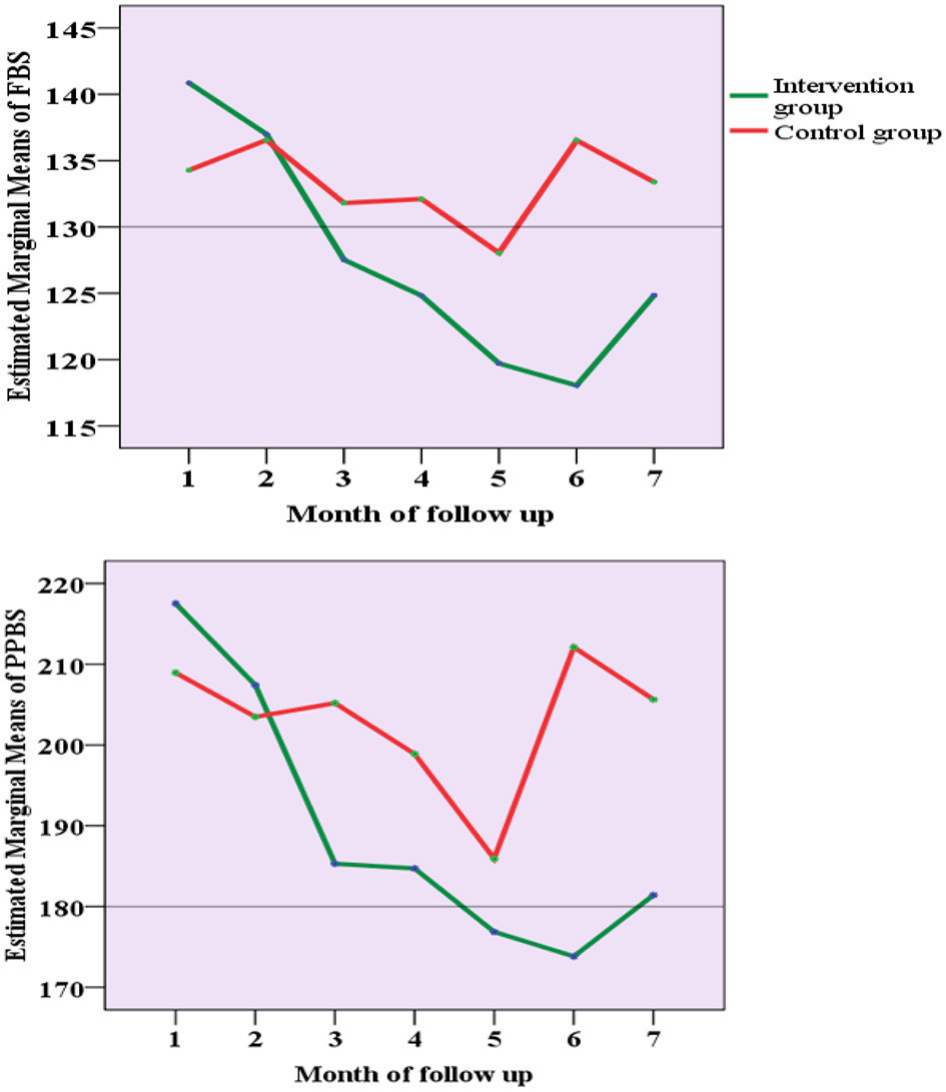
The trend of the estimated marginal mean of FBS and PPBS values over the follow-up period.

The percentage of missing values of FBS across the 6 months of follow-up ranged between 0 and 11% in the intervention group and 1.3 and 37% in the control group. The percentage of missing PPBS values ranged between 0 and 7% in the intervention group and 1.1 and 32% in the control group. Repeated measures ANOVA analysis (on the imputed data set) showed a statistically significant effect of the intervention on the FBS values over the months, *F*_(3.76,457.3)_ = 11.9, *p* < 0.001. A similar effect of the intervention on the PPBS values over 6 months was noted *F*_(3.77,539.4)_ =11.45, *p* < 0.001 (Figure 3).

### Cluster Adjusted Analysis

On adjusting for clustering at the HSC level with time, the intervention was associated with a significant decline in FBS values, with an estimated decline of 3.09 (SE = 0.57) mg/dl with each month of follow-up, *p* < 0.001. A similar cluster adjustment on PPBS values showed a 6.35 (SE = 1.04) mg/dl decline of PPBS values with intervention-time interaction compared to the control group, *p* < 0.001. The ICC calculated for FBS and PPBS values at the HSC level was 0.058 (95% CI: 0.012–0.23) and 0.048 (95% CI: 0.008–0.23), respectively. At the patient level, ICC was 0.601 (95% CI: 0.528–0.671) for FBS and 0.606 (95% CI: 0.53–0.671) for PPBS model.

The model was further adjusted for sociodemographic and baseline parameters, and the intervention-time interaction was found to reduce FBS values by 3.1 (SE = 0.57) mg/dl (*p* < 0.001). In addition, with each year since the diagnosis of diabetes, a 0.81 (SE=0.3) mg/dl increase in the FBS value was shown by the model. A similar hierarchical model for PPBS showed intervention being associated with 6.3 (SE = 1.03) mg/dl reduction of PPBS with every month of follow-up, *p* < 0.001. Years since diagnosis of diabetes (*B* = 1.5, SE = 0.59, and *p* = 0.009), diagnosed hyperlipidemia (*B* = 14.3, SE = 7.19, and *p* = 0.046) and baseline BMI (*B* = 2.04, SE = 0.87, and *p* = 0.019) values were found to be significantly associated with PPBS values. The ICC calculated for the adjusted FBS and PPBS models at the patient level were 0.54 (SE = 0.039) and 0.54 (SE = 0.037), respectively.

### Improvement in Other Clinical Parameters

The intervention was associated with a significant change in the systolic and diastolic blood pressure values at the end of follow-up after controlling for the effect of baseline blood pressure values, *F*_(1,130)_ = 23.9, *p* < 0.001 and *F*_(1,130)_ = 15.27, *p* < 0.001, respectively. No significant difference was observed in the Body Mass Index values (Table 3).

**Table 3:** Change in clinical parameters adjusted for baseline values.

**Variables**	**Group**	**Mean value at the**	**Adjusted**	**Standard**	**95% Confidence**	**Significance 2**
		**end of 6 months (SD)**	**Mean**	**error**	**interval**	**tailed** ***p***
FBS (mg/dl)	Intervention	125 ± 16	123.7	1.71	120.3 to 127.1	<0.001
	Control	134 ± 24	135.4	1.89	131.6 to 139.1	
PPBS (mg/dl)	Intervention	182 ± 42	179.7	3.59	172.6 to 186.8	<0.001
	Control	207 ± 49	210.1	3.97	202.3 to 217.9	
BMI (kg/m^2^)	Intervention	25 ± 4	25.15	.06	25.02 to 25.29	0.08
	Control	26 ± 3	25.33	0.07	25.18 to 25.47	
Systolic BP (mmHg)	Intervention	125 ± 9	125.5	1.24	123.0 to 127.9	<0.001
	Control	135 ± 14	134.5	1.37	131.8 to 137.2	
Diastolic BP (mmHg)	Intervention	80 ± 5	80.2	0.75	78.8 to 81.7	<0.001
	Control	85 ± 8	84.6	0.83	83.0 to 86.2	

### Change in the Health-Related Behaviors

The mean number of follow-up visits attended by the participants was 5.8 ± 0.5 and 4.8 ± 1 in the intervention and control groups, respectively. The difference was significant with *t* = 6.4, *p* < 0.001. While the median drug adherence score remained at 1 (1–3) throughout the months of follow-up in the control group, the score improved to 3 (3, 4) in the intervention group. GEE showed a significant change with β = 0.67, Wald chi-square = 8.0, degree of freedom = 1, *p* = 0.005.

After categorizing the patients into poor, moderate, and good drug adherence based on their medication adherence scores, the intervention group had 6 (8.3%) participants with poor drug adherence and 33 (45.8%) participants each with moderate and good drug adherence. In the control group, the proportion of participants with poor, moderate, and good drug adherence were 50.8, 30.5, and 18.6%, respectively. A significant association of intervention with drug adherence was noted, *p* < 0.001. On regrouping the participants into two categories (poor adherence and moderate-high adherence), the control group showed a 1.9 (1.5–2.4) times higher risk of poor drug adherence.

When adjusted for baseline values, the mean hours of physical activity per week was 3.6 ± 0.07 h in the intervention group and 3.0 ± 0.08 h in the control group, *F*_(1,130)_ = 9.36, *p* = 0.003. WHO-recommended adequacy of physical activity was reported only by 16 (22.2%) participants in the intervention group and 10 (16.7%) participants in the control group.

At the baseline, choosing walking as a method of traveling a kilometer distance was preferred by 38 (52.8%) and 40 (66.7%) participants in the intervention and control group, respectively, and this proportions climbed to 86.1% in the intervention group and 69.5% in the control group by the end of 6 months (*p* = 0.02). The number of participants with a self-reported habit of alcoholism remained the same in the control group, whereas three among the six alcoholics reported having stopped the habit of alcohol intake in the intervention group. Among the smokers, one person each from the intervention and control group reported quitting the habit of smoking. A significant reduction in the number of cigarettes smoked per day (*p* = 0.03) and the amount of alcohol consumption per day (*p* = 0.002) was noted in the intervention group adjusting for the baseline values.

Dietary habits at the end of follow-up were assessed after adjusting for the baseline values using one-way ANCOVA. Significant dietary changes between the intervention and control group are shown in Table 4. By the end of follow-up, 24 (33.3%) participants in the intervention group and 9 (15.3) participants in the control group had a vegetable garden at home (*p* = 0.02). No significant change was observed in initiating a fruit garden at home.

**Table 4:** Dietary habits at end of follow-up adjusting for the baseline values.

**Variables**	**Group**	**Adjusted**	**Standard**	**95% Confidence**	**Significance 2**
		**Mean**	**error**	**Interval**	**tailed p**
Sugar use (grams/day)	Intervention	10.6	0.26	10.1 to 11.2	0.001
	Control	12.0	0.29	11.4 to 12.6	
Fried snacks (days/week)	Intervention	0.59	0.07	0.44 to 0.73	<0.001
	Control	1.3	0.08	1.14 to 1.4	
Sugary drinks (days/week)	Intervention	0.07	0.04	−0.004 to 0.151	<0.001
	Control	0.37	0.04	0.28 to 0.45	
Pickle/pappad (days/week)	Intervention	1.04	0.09	0.87 to 1.21	<0.001
	Control	1.8	0.09	1.6 to 1.98	
Pulses (days/week)	Intervention	2.7	0.06	2.64 to 2.87	0.013
	Control	2.5	0.06	2.40 to 2.66	
Fruits (grams/week)	Intervention	120.6	4.6	111.4 to 129.8	0.022
	Control	104.5	5.1	94.4 to 114.7	
Green leafy vegetables (grams/week)	Intervention	115.6	4.25	107.2 to 124.0	<0.001
	Control	85.0	4.7	755.7 to 94.3	
Other vegetables (grams/week)	Intervention	405.2	6.0	393.4 to 417.0	0.033
	Control	386	6.6	372.9 to 399.0	

### Role of Individual Factors in the Intervention Package

A General Linear model was built with GEE to study the effect of factors in the intervention package on glycemic control adjusting for the sociodemographic variables, and baseline values of drug adherence, dietary habits, physical activity, alcoholism, and smoking frequency. The model showed drug adherence score (*p* < 0.001), physical activity in hours per week (*p* < 0.001), Body Mass Index (*p* = 0.002), fruit intake (*p* = 0.004), and green leafy vegetable intake (*p* = 0.01) as the major predictors of FBS control (Table 5).

**Table 5:** Factors associated with glycemic control.

**Variables**	**FBS control**			**PPBS control**		
	***B***	Wald chi-square^*^	***p*** **-value**	***B***	Wald chi-square^*^	***P-*** **value**
Drug adherence score	0.4	26.5	<0.001	0.59	27.9	<0.001
Physical activity (hours per week)	0.06	13.9	<0.001	0.21	4.6	0.03
Body Mass Index (kg/m^2^)	0.1	9.7	0.002	0.14	8.5	0.005
Fruit intake (in grams per week)	0.01	8.5	0.004	0.01	0.11	0.7
Green leafy vegetable intake (in grams per week)	0.01	6.3	0.01	0.01	4.7	0.03

* *Degree of freedom = 1*.

*The corrected Quasi Likelihood under the independence Model Criterion (QICC) was 633 and 263 for the FBS and PPBS model, respectively*.

### Improvement in Service Delivery at the HSCs

Adjusting for the baseline values, the practice/skills score of the JPHNs in the intervention group was 18.2 (95% CI: 16.9–19.4) and in the control group was 7.6 (95% CI: 6.2–8.9), the difference being significant at *p* < 0.001. No significant difference was noted in the knowledge score. At the end of follow up 65 (90.3%) participants in the intervention group reported the quality of services received from their HSC to be good (an increase from a baseline value of 37%) and the remaining reported it to be satisfactory. In the control group, 10 (16.9%), 45 (76.3%), and 4 (6.8%) participants reported the services as good, satisfactory, and poor, respectively. As per the participants, there was a major difference in the counseling services received in each group, with almost all patients in the intervention group having received counseling on a healthy diet, drug adherence, and regular follow-up.

## Discussion

The intervention was successful in reducing the Fasting and Postprandial blood glucose of participants, improving their glycemic control and its maintenance. Non-pharmacological interventions have been shown to improve knowledge, health behavior, and glycemic control among diabetics and prediabetics across the globe (13, 20–26). However, there are pragmatic primary care trials that failed to show significant results (27–29). Most studies emphasize the role of nutrition, exercise, and the importance of diabetes self-care including medication adherence and routine follow-up. Evidence of a community-based participatory learning approach in diabetes prevention and management with the help of Community Health Workers was documented in the Indian population as early as 2012 (30). Even after the demonstration of many successful models worldwide, the NCD control statistics fall far below the desired level (31). The complexity including resource intensity and individualized approaches are hindrances behind the implementation. Further, most of the studies were done in academic centers and their emulation at a community level is grueling. Our study is the first randomized controlled trial from India to demonstrate the effectiveness of a community health worker/ multipurpose health worker-led intervention integrated into the health system for diabetes management at the primary care level. Such an intervention can be pivotal as a sustainable tool at the community level.

In this study, the mean fasting blood sugar values showed a reduction of 16 mg/dl in the intervention group whereas no notable difference was observed in the control group. A Community Health Worker based integrated approach to cardiovascular risk reduction from India reported 43.0 ± 83.5 mg/dl FBS reduction in the intervention group and 16.3 ± 77.2 mg/dl reduction in the control group with 2 years of follow up (32). A recent systematic review and meta-analysis on community-based programs for diabetes prevention reported a significant reduction in FBS values along with BMI and waist circumference, however, a significant difference was not demonstrated in 2-h PPBS values and blood pressure (33). The Kerala Diabetes Prevention Programme using a community-based peer support lifestyle intervention showed a non-significant reduction in the incidence of diabetes with significant improvement in some cardiovascular factors (34). Besides glycemic control, the present intervention could bring a significant reduction in blood pressure values of participants but not in the BMI. A significant reduction in BMI may require a more intensive intervention strategy or a longer duration of follow-up (35). An intervention study through Community Health Workers among hypertensive patients in a rural community in Kerala demonstrated significant improvement (36).

The study population represented NCD primary care utilization in LMICs with the predominance of elderly, females, and lower-middle-class population. Gender differences in the utilization of primary health services were reported by many studies across the globe (13, 37, 38). The poor baseline diabetes control status in both groups is comparable to the findings reported from the country in nationwide surveys including the ICMR-INDIAB study and the TIGHT study (3, 39). Chronic diseases like diabetes require good patient motivation for long-term therapy adherence, and suboptimal therapy can hinder the control and engender complications. Moreover, Diabetes is difficult to monitor and control compared with hypertension and requires more active self-management by the patients (26). The baseline adherence rates observed in this study is comparable to the WHO estimates of <50% adherence to long-term therapy in LMICs (40).

Though changes in health-related behaviors of the patient and physician behaviors were found to be possible mechanisms of improved glycemic control in many studies, the role of improved medication adherence was hypothesized to be the major contributor (25, 41, 42). In our trial, medication adherence and the number of follow-ups attended showed the most significant improvement. A remarkable upshot of the intervention was a significant increase in the preference of walking a distance of one kilometer instead of using a public or private vehicle and a reduction in the intake of unhealthy food items. Despite a significant increase in the consumption of fruits and vegetables, none of the study participants reported an adequate intake. A pragmatic trial from rural Kerala through neighborhood groups highlighted the difficulty in increasing the fruit and vegetable intake in the community and the need to explore new strategies (43). Due to the nature of the family system in India, explaining a dietary change at the family level requires special attention along with administrative level actions like ensuring the availability of healthy foods and stabilization of prices (24, 44, 45).

Despite the effectiveness of educational interventions in diabetes management, a major challenge across populations is the maintenance of glycemic control (46, 47). We tried to generate evidence on the maintenance of glycemic control through a 6-month follow-up of participants. Sustainability is the arduous part of any lifestyle change intervention and can be made feasible only with system changes, community efforts, and peer reinforcement. The role of social support groups in the maintenance of glycemic control was studied by Ing et al. (46) However, it is not always feasible to involve additional manpower in a resource-poor setting. Our study was a pilot to create a system change at the primary care level involving community participation. Better health worker attitude and skills could bring forth satisfaction and a better understanding of disease control among the patients. Continuance of the lifestyle changes was further ensured through simple measures like vegetable/fruit garden in the house, walking groups, and dairy maintenance. The program can serve as a scalable low-cost model for low-middle income countries in the control of chronic diseases.

International evidence has shown insufficient training and skills to be the major loophole behind the failure of CHW programs (11, 23). Our trial put forth an insight into the gaps in the training process of the health staff at the primary care level. Though the staff had adequate knowledge on diabetes care and non-pharmacological interventions, they failed to demonstrate the necessary attitude and skill in patient counseling which could be improved by the intervention provided. Every training mechanism should target the affective and psychomotor domains of the participants using non-traditional training methods like group discussions, role-play, hands-on training, etc. along with the cognitive domain. The study evaluated the training using all levels of Miller's pyramid, unlike most studies that tried to measure improvement in knowledge. A World Health Organization, 2003 report on medication adherence observed that a negative attitude of nurses and paramedical staff is responsible for 50% of poor drug adherence among patients (40). Positive attitude and commitment of health staff is not a choice, but a necessity. Attitude building, regular supervision and feedback, non-traditional training methods, and multifaceted interventions can bring a change (48).

The study is a novel attempt to incorporate training on non-pharmacological interventions which can reflect an attitude change among the field-level health workers in the existing primary health system. The minimal loss to follow-up may be attributed to the integration into the preexisting health system. Our study has some limitations. The study was conducted in a limited setting under a medical teaching institution. However, the setting resembles the peripheral health care institutes throughout the state. A major limitation is the possibility of measurement error because of the self-reporting nature of the health-related habits, vulnerable to social desirability. Considering the sustainability of the intervention, the 6-month evaluation period is relatively short and needs further follow-up assessments. We used Fasting and Postprandial blood glucose as a measure of glycemic control instead of HbA1c (which could have been a more robust measure), as the frequency of HbA1c monitoring is poor in our setting and the former may be a more sustainable outcome measure in a pragmatic setting. Moreover, there is increasing recognition of PPBS as an important measure of the overall glycemic burden and a more reliable predictor of cardiovascular disease complications (49, 50). Since the study was done on a small sample, an imbalance of some baseline variables was noted between the two groups and we have tried to adjust for this difference in our analysis. However, a possibility of residual confounding by measured and unmeasured variables cannot be excluded. Since the exact intraclass correlation was not known, our sample size calculation involved inflation of sample size by 10% instead of calculating the design effect. But, we have calculated the ICC from the study data and have adjusted for clustering in the analysis. There exists a possibility of some degree of contamination. This was minimized by informing the intervention group health workers not to discuss the information in the training module with their colleagues throughout the trial period.

## Conclusion

This trial data brings experimental evidence to the effectiveness of a non-pharmacological intervention through field-level health workers which can be incorporated into the existing health system to improve and maintain glycemic control among diabetic patients in the primary care settings. The study has shown that a well-designed health worker intervention can translate to attitude change and skill development which in turn can reflect in the improvement of glycemic control among the patients. The study emphases the need to reorient training in the health sector to address the affective and psychomotor domain through non-traditional training methods. The study also attempted to elucidate the mechanism by which the intervention improved glycemic control of which improvement in drug adherence, follow-up, physical activity, fruit and vegetable intake by the participants were found to be the major contributors. Despite demonstrating the role of diet and physical activity, the achievement of adequacy in these regards remained very low. More studies need to be planned to incorporate better interventions in the health system.

### Recommendations

Task sharing interventions involving counseling on drug adherence, regular follow-up, healthy diet involving fruits and vegetables, adequate physical activity, and smoking and alcohol cessation should become an integral part of non-communicable disease management at the primary care level. Multifaceted health worker interventions including non-traditional training methods and attitude building can help tackle the growing burden of NCDs in low and middle-income countries. Training programs for health workers should be meticulously designed and tested for efficiency. Moreover, every training program should be evaluated for immediate and long-term effects. Researches should be designed to identify barriers to behavior change so that new strategies can be identified in developing and implementing self-management plans for diabetic patients. This includes the identification of decision-making dynamics in diet and physical activity of individuals and families. Specific health system interventions with intersectoral cooperation need to be planned in this regard.

## Data Availability Statement

The raw data supporting the conclusions of this article will be made available by the authors, without undue reservation.

## Ethics Statement

The studies involving human participants were reviewed and approved by Human Ethics Committee, Government Medical College, Thiruvananthapuram. Ethics clearance number: IEC No. 12/01/2017/MCT. The patients/participants provided their written informed consent to participate in this study.

## Author Contributions

AR was the principal investigator, responsible for the oversight of the study, drafted the manuscript, and swears the manuscript to be honest and transparent. AR, SC, TA, and PI contributed to the study design. AR and KP created data collection tools and led data collection. AR, TA, and KP conducted the data analysis and produced figures and tables. The critical review of the manuscript was done by PI, SC, and TA. All authors participated in the interpretation of the results and had full access to the data and affirm integrity.

## Conflict of Interest

The authors declare that the research was conducted in the absence of any commercial or financial relationships that could be construed as a potential conflict of interest.

## Publisher's Note

All claims expressed in this article are solely those of the authors and do not necessarily represent those of their affiliated organizations, or those of the publisher, the editors and the reviewers. Any product that may be evaluated in this article, or claim that may be made by its manufacturer, is not guaranteed or endorsed by the publisher.

## References

[1] Mohan V Sandeep S Deepa R Shah B Varghese C. Epidemiology of type 2 diabetes: Indian scenario. Indian J Med Res. (2007) 125:217–30. 17496352

[2] NCD - Non Communicable Diseases Control Programme. National Health Mission. Available online at: http://arogyakeralam.gov.in/2020/03/23/ncdnon-communicable-diseases-control-programme/ (cited March 27, 2021).

[3] Borgharkar SS Das SS. Real-world evidence of glycemic control among patients with type 2 diabetes mellitus in India: the TIGHT study. BMJ Open Diabetes Res Care. (2019) 7:e000654. 10.1136/bmjdrc-2019-000654 31413840 PMC6673766

[4] Soman SK Areekal B Sukumaran ST Puliyakkadi S Ravi RK. Poor-glycaemic-control prevalence and determinants among type 2 diabetes mellitus patients attending a primary health care setting in central Kerala. J Evid Based Med Healthc. (2020) 7:2892–7. 10.18410/jebmh/2020/592

[5] Kumar SP Sandhya A. A study on the glycemic, lipid and blood pressure control among the type 2 diabetes patients of north Kerala, India. Indian Heart J. (2018) 70:482–5. 10.1016/j.ihj.2017.10.007 30170640 PMC6116718

[6] Kruk ME Porignon D Rockers PC Van Lerberghe W. The contribution of primary care to health and health systems in low- and middle-income countries: a critical review of major primary care initiatives. Soc Sci Med. (2010) 70:904–11. 10.1016/j.socscimed.2009.11.025 20089341

[7] Palmas W March D Darakjy S Findley SE Teresi J Carrasquillo O. Community health worker interventions to improve glycemic control in people with diabetes: a systematic review and meta-analysis. J Gen Intern Med. (2015) 30:1004–12. 10.1007/s11606-015-3247-0 25735938 PMC4471021

[8] Smalls BL Walker RJ Bonilha HS Campbell JA Egede LE. Community interventions to improve glycemic control in african americans with type 2 diabetes: a systemic review. Glob J Health Sci. (2015) 7:171–82. 10.5539/gjhs.v7n5p171 26156923 PMC4803865

[9] Norris SL Chowdhury FM Le KV Horsley T Brownstein JN Zhang X. Effectiveness of community health workers in the care of persons with diabetes. Diabet Med. (2006) 23:544–56. 10.1111/j.1464-5491.2006.01845.x 16681564

[10] Mitchell LJ Ball LE Ross LJ Barnes KA Williams LT. Effectiveness of dietetic consultations in primary health care: a systematic review of randomized controlled trials. J Acad Nutr Diet. (2017) 117:1941–62. 10.1016/j.jand.2017.06.364 28826840

[11] Hill J Peer N Oldenburg B Kengne AP. Roles, responsibilities and characteristics of lay community health workers involved in diabetes prevention programmes: a systematic review. PLoS One. (2017) 12:e0189069. 10.1371/journal.pone.0189069 29216263 PMC5720739

[12] Boutron I Altman DG Moher D Schulz KF Ravaud P DJ C. CONSORT statement for randomized trials of nonpharmacologic treatments: a 2017 update and a CONSORT extension for nonpharmacologic trial abstracts. Ann Intern Med. (2017). 167:40–7. 10.7326/M17-0046 28630973

[13] Goldhaber-Fiebert JD Goldhaber-Fiebert SN Tristán ML Nathan DM. Randomized controlled community-based nutrition and exercise intervention improves glycemia and cardiovascular risk factors in type 2 diabetic patients in rural Costa Rica. Diabetes Care. (2003) 26:24–9. 10.2337/diacare.26.1.24 12502654

[14] Custer RL Scarcella JA Stewart BR. The modified delphi technique - a rotational modification. J Career Tech Educ. (1999) 15:50–8. 10.21061/jcte.v15i2.702

[15] DIABETES-MELLITUS-TREATMENT-PROTOCOL.pdf. Available online at: https://dhs.kerala.gov.in/wp-content/uploads/2021/01/DIABETES-MELLITUS-TREATMENT-PROTOCOL.pdf (accessed August 25, 2021).

[16] Association AD. 6. Glycemic targets: standards of medical care in diabetes-2019. Diabetes Care. (2019) 42(Suppl. 1):S61–S70. 10.2337/dc19-S006 30559232

[17] WHO. Physical activity. Available online at: https://www.who.int/news-room/fact-sheets/detail/physical-activity (accessed March 10, 2021).

[18] WHO Expert Consultation. Appropriate body-mass index for Asian populations and its implications for policy and intervention strategies. Lancet Lond Engl. (2004) 363:157–63. 10.1016/S0140-6736(03)15268-3 14726171

[19] Morisky DE Green LW Levine DM. Concurrent and predictive validity of a self-reported measure of medication adherence. Med Care. (1986) 24:67–74. 10.1097/00005650-198601000-00007 3945130

[20] Alaofè H Asaolu I Ehiri J Moretz H Asuzu C Balogun M. Community health workers in diabetes prevention and management in developing countries. Ann Glob Health. (2017) 83:661–75. 10.1016/j.aogh.2017.10.009 29221543

[21] Lawlor MS Blackwell CS Isom SP Katula JA Vitolins MZ Morgan TM. Cost of a group translation of the Diabetes Prevention Program: Healthy Living Partnerships to Prevent Diabetes. Am J Prev Med. (2013) 44(4 Suppl. 4):S381–S9. 10.1016/j.amepre.2012.12.016 23498303 PMC3839056

[22] Forouhi NG Misra A Mohan V Taylor R Yancy W. Dietary and nutritional approaches for prevention and management of type 2 diabetes. BMJ. (2018) 361:k2234. 10.1136/bmj.k2234 29898883 PMC5998736

[23] Ackermann RT Finch EA Brizendine E Zhou H Marrero DG. Translating the diabetes prevention program into the community the DEPLOY pilot study. Am J Prev Med. (2008) 35:357–63. 10.1016/j.amepre.2008.06.035 18779029 PMC2610485

[24] Islam NS Zanowiak JM Wyatt LC Kavathe R Singh H Kwon SC. Diabetes prevention in the New York City Sikh Asian Indian Community: a Pilot Study. Int J Environ Res Public Health. (2014) 11:5462–86. 10.3390/ijerph110505462 24852392 PMC4053907

[25] Newman PM Franke MF Arrieta J Carrasco H Elliott P Flores H. Community health workers improve disease control and medication adherence among patients with diabetes and/or hypertension in Chiapas, Mexico: an observational stepped-wedge study. BMJ Glob Health. (2018) 3:e000566. 10.1136/bmjgh-2017-000566 29527344 PMC5841495

[26] Ndou T van Zyl G Hlahane S Goudge J. A rapid assessment of a community health worker pilot programme to improve the management of hypertension and diabetes in Emfuleni sub-district of Gauteng Province, South Africa. Glob Health Action. (2013) 6:19228. 10.3402/gha.v6i0.19228 23364086 PMC3556684

[27] Edelman D Dolor RJ Coffman CJ Pereira KC Granger BB Lindquist JH. Nurse-led behavioral management of diabetes and hypertension in community practices: a randomized trial. J Gen Intern Med. (2015) 30:626–33. 10.1007/s11606-014-3154-9 25567758 PMC4395596

[28] Liss DT Finch EA Cooper A Sheth A Tejuosho AD Lancki N. One-year effects of a group-based lifestyle intervention in adults with type 2 diabetes: a randomized encouragement trial. Diabetes Res Clin Pract. (2018) 140:36–44. 10.1016/j.diabres.2018.03.030 29596950 PMC5990453

[29] Browning C Chapman A Yang H Liu S Zhang T Enticott JC. Management of type 2 diabetes in China: the Happy Life Club, a pragmatic cluster randomised controlled trial using health coaches. BMJ Open. (2016) 6:e009319. 10.1136/bmjopen-2015-009319 26944692 PMC4785304

[30] Balagopal P Kamalamma N Patel TG Misra R. A community-based participatory diabetes prevention and management intervention in rural India using community health workers. Diabetes Educ. (2012) 38:822–34. 10.1177/0145721712459890 23033123

[31] World Health Organization. Global Status Report on Non-communicable Diseases 2010 (2011). Available online at: https://apps.who.int/iris/handle/10665/44579 (accessed March 30, 2021).

[32] Khetan A Zullo M Rani A Gupta R Purushothaman R Bajaj NS. Effect of a community health worker-based approach to integrated cardiovascular risk factor control in india: a cluster randomized controlled trial. Glob Heart. (2019) 14:355–65. 10.1016/j.gheart.2019.08.003 31523014 PMC7358973

[33] Shirinzadeh M Afshin-Pour B Angeles R Gaber J Agarwal G. The effect of community-based programs on diabetes prevention in low- and middle-income countries: a systematic review and meta-analysis. Glob Health. (2019) 15:10. 10.1186/s12992-019-0451-4 30709362 PMC6359819

[34] Thankappan KR Sathish T Tapp RJ Shaw JE Lotfaliany M Wolfe R. A peer-support lifestyle intervention for preventing type 2 diabetes in India: a cluster-randomized controlled trial of the Kerala Diabetes Prevention Program. PLOS Med. (2018) 15:e1002575. 10.1371/journal.pmed.1002575 29874236 PMC5991386

[35] Flodgren G Gonçalves-Bradley DC Summerbell CD. Interventions to change the behaviour of health professionals and the organisation of care to promote weight reduction in children and adults with overweight or obesity. Cochrane Database Syst Rev. (2017) 11:CD000984. 10.1002/14651858.CD000984.pub3 29190418 PMC6486102

[36] Thankappan KR Sivasankaran S Mini GK Daivadanam M Sarma PS Abdul Khader S. Impact of a community based intervention program on awareness, treatment and control of hypertension in a rural Panchayat, Kerala, India. Indian Heart J. (2013) 65:504–9. 10.1016/j.ihj.2013.08.023 24206872 PMC3861297

[37] Bertakis KD Azari R Helms LJ Callahan EJ Robbins JA. Gender differences in the utilization of health care services. J Fam Pract. (2000) 49:147–52. 10718692

[38] Barreto SM Kalache A Giatti L: Does health status explain gender dissimilarity in health care use among older adults? Cad Saude Publ. (2006) 22:347–55. 10.1590/S0102-311X2006000200012 16501747

[39] Unnikrishnan R Anjana RM Deepa M Pradeepa R Joshi SR Bhansali A. Glycemic control among individuals with self-reported diabetes in india-the ICMR-INDIAB study. Diabetes Technol Ther. (2014) 16:596–603. 10.1089/dia.2014.0018 25101698 PMC4135327

[40] Sabaté E editor. Adherence to Long-Term Therapies: Evidence for Action. Geneva: World Health Organization (2003).

[41] Shaji S Rajendran D Kumpatla S Viswanathan V. Evaluation of diabetes self-care with self-monitoring of blood glucose among type 2 diabetic patients and its impact on HbA1c. Int J Diabetes Dev Ctries. (2013) 33:181–2. 10.1007/s13410-013-0118-2

[42] Rhee MK Slocum W Ziemer DC Culler SD Cook CB El-Kebbi IM. Patient adherence improves glycemic control. Diabetes Educ. (2005) 31:240–50. 10.1177/0145721705274927 15797853

[43] Daivadanam M Wahlström R Ravindran TKS Sarma PS Sivasankaran S Thankappan KR. Changing household dietary behaviours through community-based networks: a pragmatic cluster randomized controlled trial in rural Kerala, India. PLoS One. (2018) 13:e201877. 10.1371/journal.pone.0201877 30133467 PMC6104953

[44] Ayantunji G. Exploring the Dynamics of Consumerism in Developing Nations. IGI Global (2019). 451 p.

[45] Frank SM Webster J McKenzie B Geldsetzer P Manne-Goehler J Andall-Brereton G. Consumption of fruits and vegetables among individuals 15 years and older in 28 low- and middle-income countries. J Nutr. (2019) 149:1252–9. 10.1093/jn/nxz040 31152660

[46] Ing CT Zhang G Dillard A Yoshimura SR Hughes C Palakiko D-M. Social support groups in the maintenance of glycemic control after community-based intervention. J Diabetes Res. (2016) 2016:7913258. 10.1155/2016/7913258 27563680 PMC4987463

[47] Norris SL Lau J Smith SJ Schmid CH Engelgau MM. Self-management education for adults with type 2 diabetes: a meta-analysis of the effect on glycemic control. Diabetes Care. (2002) 25:1159–71. 10.2337/diacare.25.7.1159 12087014

[48] Rowe AK de Savigny D Lanata CF Victora CG. How can we achieve and maintain high-quality performance of health workers in low-resource settings? Lancet Lond Engl. (2005) 366:1026–35. 10.1016/S0140-6736(05)67028-6 16168785

[49] Sorkin JD Muller DC Fleg JL Andres R. The relation of fasting and 2-h postchallenge plasma glucose concentrations to mortality: data from the Baltimore Longitudinal Study of Aging with a critical review of the literature. Diabetes Care. (2005) 28:2626–32. 10.2337/diacare.28.11.2626 16249530

[50] Meigs JB Nathan DM D'Agostino RB Wilson PWF Framingham Offspring Study. Fasting and postchallenge glycemia and cardiovascular disease risk: the Framingham Offspring Study. Diabetes Care. (2002) 25:1845–50. 10.2337/diacare.25.10.1845 12351489

